# Supplementary material for: Can Optic Nerve Sheath Images on a Thin-Slice Brain Computed Tomography Reconstruction Predict the Neurological Outcomes in Cardiac Arrest Survivors?
Source: J Clin Med. 2022 Jun 26;11(13):3677. doi: 10.3390/jcm11133677 (PMC9267811; doi:10.3390/jcm11133677)
Supplement: Supplementary file 1 [file jcm-11-03677-s001.zip › jcm-1704478-supplementary.pdf]

Supplementary Materials

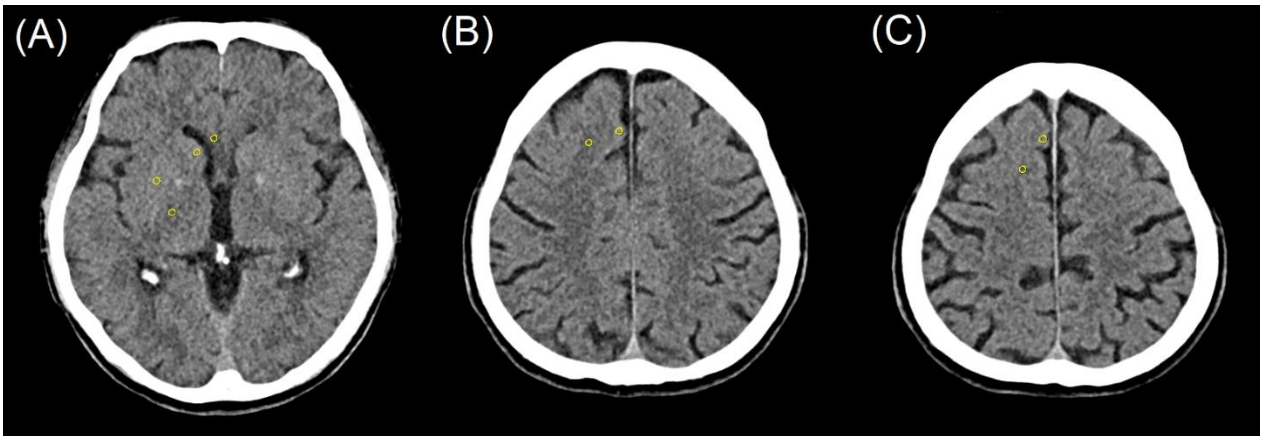

Figure S1. Brain computed tomography images showing the measurements in Hounsfield units. The circular regions of interest (10 mm^2) are positioned in the caudate nucleus (CN), putamen (PU), posterior limb of the internal capsule (PLIC), and genu of the corpus callosum (CC) at the level of the basal ganglia (A). Gray and white matter from the medial cortex was measured at the centrum semiovale (MC1, MW1) (B) and high cortical level (MC2, MW2) (C).

The gray-to-white matter ratios (GWRs) were calculated according to the following formulas:

GWR Basal ganglia = (CN + PU)/(CC + PLIC)

GWR Cerebrum = (MC1 + MC2)/(MW1 +MW2)

GWR Average = (GWR Basal ganglia +GWR Cerebrum)/2

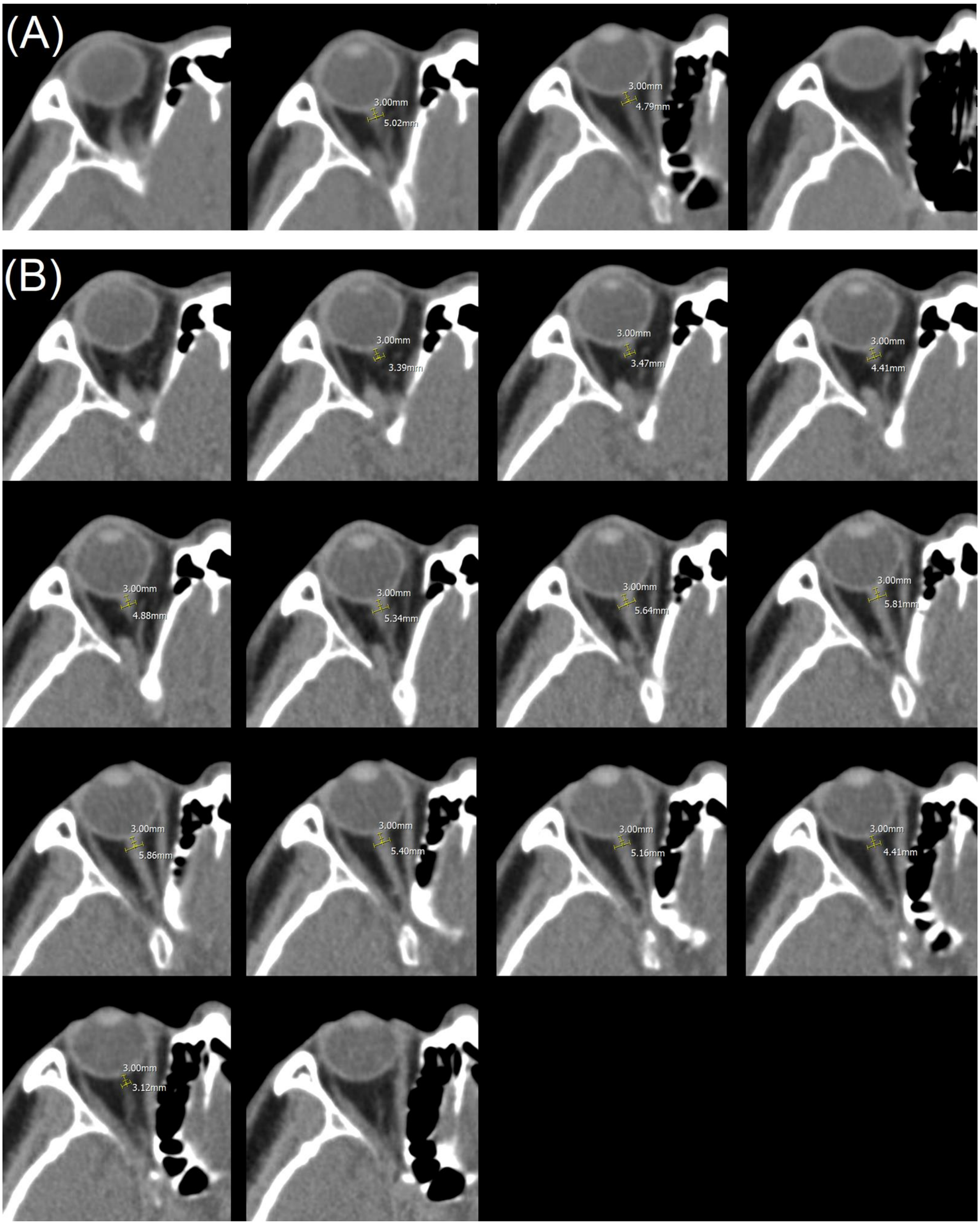

Figure S2. Consecutive optic nerve sheath images of a 62-year-old male cardiac arrest survivor. (A) On 4 mm routine-slice brain computed tomography images, the optic nerve sheath was observed on only 2 axial images, and the routine optic nerve sheath diameter was measured as 5.02 mm. (B) On 0.6 mm thin-slice images, the optic nerve sheath was observed in 12 axial images, and the maximal optic nerve sheath diameter was measured as 5.86 mm.

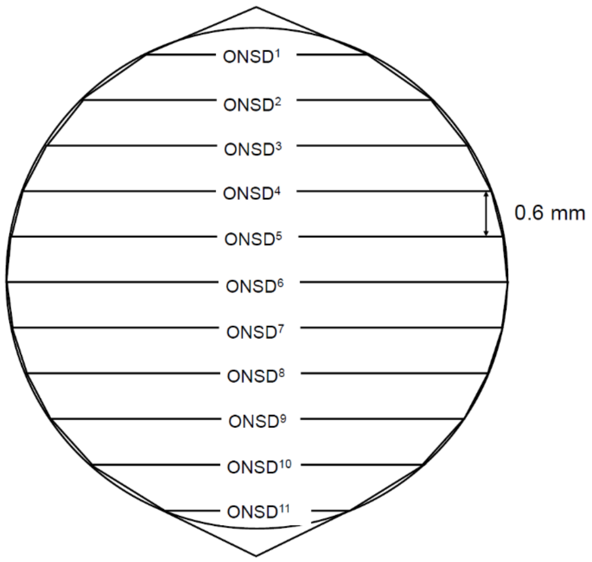

Figure S3. The optic nerve sheath area. It was calculated from all of the measured optic nerve sheath diameters on each 0.6 mm-slice axial image according to the following formula:

ONSA = $[1.5\times ONSD^1 + 2\times (ONSD^2 + ONSD^3 \ldots . ONSD^9 + ONSD^{10}) + 1.5\times ONSD^{11}] \times 0.6/2\ mm^2$

ONSA, optic nerve sheath area; ONSD, measured optic nerve sheath diameter.

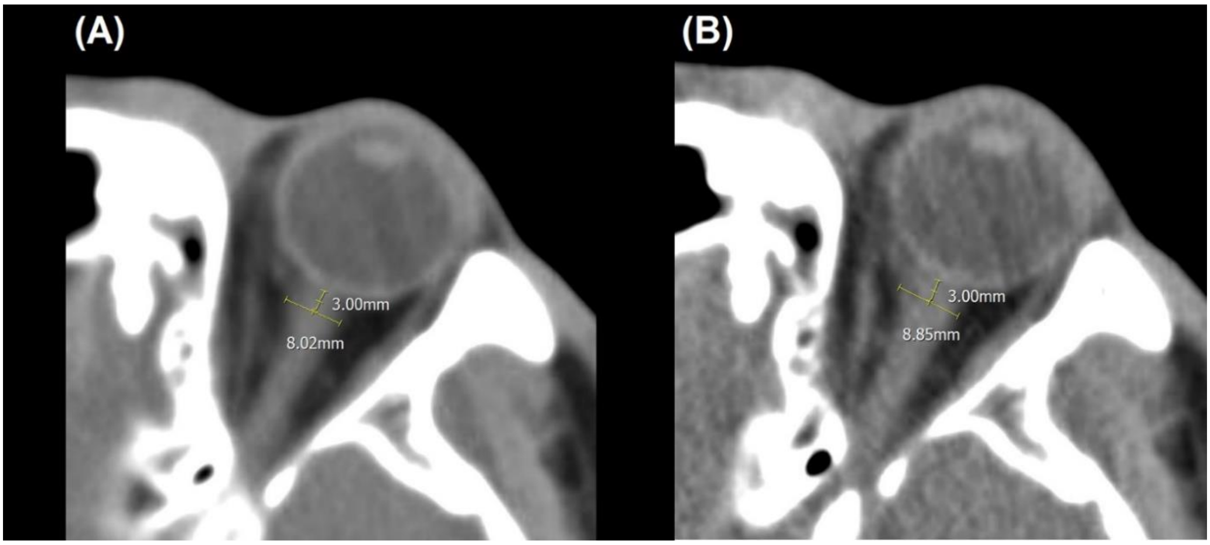

Figure S4. Optic nerve sheath images of a 43-year-old male cardiac arrest survivor. The optic nerve sheath diameter of the left optic nerve was measured as 8.02 mm from the 4 mm routine-slice computed tomography (CT) image (A) and 8.85 mm from the 0.6 mm thin-slice CT image (B). The patient regained consciousness immediately after rewarming and had a cerebral performance category of 1 at 6 months after the cardiac arrest.
